# Sustaining the General Practice Nursing Workforce in Australia: Demographics, Job Satisfaction, and Professional Development Challenges

**DOI:** 10.1111/nhs.70130

**Published:** 2025-05-12

**Authors:** Wenpeng You, Claire Verrall, Eileen Willis, Danny Hills

**Affiliations:** ^1^ School of Biomedicine, The University of Adelaide Adelaide Australia; ^2^ School of Nursing and Midwifery, Western Sydney University Sydney Australia; ^3^ Adelaide Nursing School, The University of Adelaide Adelaide Australia; ^4^ College of Nursing and Health Sciences, Flinders University Adelaide Australia; ^5^ School of Nursing, Midwifery and Social Sciences, Central Queensland University Rockhampton Australia; ^6^ Monash Nursing and Midwifery, Monash University Melbourne Australia; ^7^ Research and Innovation, Australian Primary Health Care Nurses Association Melbourne Australia

**Keywords:** general practice nurses, job satisfaction, primary heal care, professional development, workforce sustainability

## Abstract

General practice nurses (GPNs) are essential members of multidisciplinary primary care teams. Understanding their demographics, career trajectories, and professional challenges is crucial for workforce sustainability. This study examines the demographic characteristics, professional experiences, and career intentions of GPNs in Australia, focusing on workforce sustainability, job satisfaction, and professional development. A cross‐sectional survey was conducted among GPNs across Australia. Descriptive analysis, factor analysis, and logistic regression examined demographic trends and professional outcomes. The workforce is aging, with many nearing retirement and an underrepresentation of younger and culturally diverse nurses, including Aboriginal and Torres Strait Islanders. Job satisfaction was moderate, influenced by work–life balance, remuneration, and professional development. Barriers to continuing education included financial constraints and limited institutional support. Logistic regression identified employment status, pay, and professional development as key predictors of job satisfaction and retention. Sustaining the GPN workforce requires strategies to retain experienced nurses, attract younger and diverse entrants, and enhance professional development. Stable funding, an expanded scope of practice, and stronger continuing education support are essential for meeting Australia's evolving primary care needs.

**Summary**

- The aging GPN workforce, coupled with low representation of younger nurses and minorities, highlights the need for targeted recruitment and diversity initiatives.
- Financial, time, and funding challenges restrict GPNs' access to professional development, necessitating tailored education opportunities and policy reforms.
- Enhancing pay, expanding roles, and supporting work–life balance are critical for recruiting and retaining GPNs and ensuring workforce sustainability.

## 1 Introduction

Nursing is the largest health care profession globally, with nurses playing a pivotal role in delivering patient care within general practice and family medicine settings (Verrall et al. 2023). In Australia, general practice nurses (GPNs) are integral to multidisciplinary teams, working alongside general practitioners to provide essential services such as disease prevention, chronic disease management, and health promotion (Halcomb and Bird 2020). Their role is particularly vital in addressing the healthcare needs of Australia's diverse and aging population. However, a looming shortage of nurses across both acute and primary care sectors poses a significant challenge. This issue extends beyond developing nations to developed economies, including Australia, the United Kingdom, and Canada (Skillman and Toms 2022; Papp et al. 2019). The COVID‐19 pandemic has further exacerbated this situation, placing additional strain on healthcare systems and contributing to burnout among nurses. Many GPNs have adopted “quiet quitting,” where they disengage from non‐essential aspects of their roles, potentially impacting patient care and workforce sustainability (Galanis et al. 2023). In response, the Australian federal government has initiated strategies, including scope of practice reviews, funding reforms and recruitment campaigns, to tackle these workforce challenges (Kidd 2024; Reid and Knight 2024). Understanding the characteristics, experiences, and challenges of GPNs is crucial for informing these efforts, given their central role in Australia's primary healthcare system.

Extensive research has explored aspects of the nursing workforce, including age distribution, gender diversity, job satisfaction, and career progression (Skillman and Toms 2022; Ferrazzo et al. 2012). However, studies focusing specifically on GPNs remain scarce, despite their increasing significance in the Australian healthcare landscape (Halcomb and Ashley 2019; Halcomb and Ashley 2017; Halcomb et al. 2021). A notable concern is the aging workforce, which is particularly relevant to GPNs (APNA 2024). This trend, combined with an aging patient population, exacerbates the mismatch between the growing demand for healthcare services and the availability of qualified nurses (Alkan et al. 2024; Cormack 2024). To address this gap, a detailed understanding of demographic trends within the GPN workforce is essential to inform effective recruitment and retention strategies (Nicole Zonin 2022).

Cultural diversity is another critical factor for the GPN workforce. Nurses in general practice often work in multicultural communities, requiring the delivery of culturally sensitive care (Maheen et al. 2021). Despite this need, the workforce lacks adequate representation from culturally and linguistically diverse (CALD) backgrounds, including Aboriginal and Torres Strait Islander communities (Kamau et al. 2022; MacAskill et al. 2022). Enhancing cultural diversity within the GPN workforce is essential for addressing health inequities and meeting the needs of Australia's diverse population (Chenowethm et al. 2006; Khatri and Assefa 2022). Policies promoting inclusivity and equity in the workforce are crucial for fostering health equity.

Professional development is equally critical for GPNs, who maintain a broad scope of practice in general practice settings (Halcomb et al. 2021). Continuous education is essential for staying updated on advancements in medical knowledge, emerging technologies, and evolving patient needs (Mlambo et al. 2021). For GPNs, this includes managing chronic conditions, promoting preventive care, and addressing complex social and health issues (McKenna et al. 2015). Australian legislation mandates annual continuing professional development (CPD) hours to help ensure nurses maintain professional competence and meet the standards set by the Nursing and Midwifery Board of Australia (Ahpra 2024). However, many GPNs face barriers to professional development, such as financial constraints, time limitations, and lack of institutional support, especially in private general practice settings where resources are often limited (Walter and Terry 2021). The additional pressures from the COVID‐19 pandemic, including increased workloads and emotional exhaustion, have further hindered professional development efforts in this sector.

Addressing these challenges requires an understanding of how demographic trends, cultural diversity, and professional development interact within the GPN workforce. Workforce shortages, combined with rising demand for primary care services, highlight the need to enable GPNs to work to their full scope of practice, as emphasized in the Scope of Practice Review (Cormack 2024; APNA 2025a).

This study examines the demographic trends, educational profiles, professional experiences, and career intentions of GPNs in Australia. It focuses on their impact on job satisfaction, professional growth, retention, and workforce sustainability. The findings aim to guide policies that enhance recruitment, retention, and professional development, addressing workforce challenges and meeting the evolving needs of primary care in Australia.

## 2 Methods

### 2.1 Study Design

This cross‐sectional, nationwide survey in Australia aimed to generate reliable data on the General Practice Nursing and Midwifery (GPN) workforce. Such data are essential for understanding factors influencing nurses' and midwives' work decisions, satisfaction, and long‐term trends in personal, professional, and workplace factors affecting the PHC workforce.

The study used a 17‐item scale to examine job satisfaction and well‐being among GPNs. The scale combined the validated 10‐item Job Satisfaction Scale (JSS), which assesses general job satisfaction (Hills et al. 2012), with seven additional items adapted from the Medicine in Australia: Balancing Employment and Life (MABEL) survey (MABEL 2024; Joyce et al. 2010). The JSS measured general job satisfaction, while the MABEL items captured work–life balance, support, and communication, which are key aspects of primary care nursing identified through expert consultations and literature reviews (Mlambo et al. 2021; Walter and Terry 2021).

The integration of these scales reflected their shared focus on job satisfaction and well‐being. The JSS provided reliability, while the MABEL items addressed GPN‐specific factors. Expert reviews confirmed the clarity and relevance of all items, supporting the scale's application in this study.

To understand the levels of GPNs' job satisfaction and well‐being, descriptive analysis was conducted for each of the 17 items before further examination. Differences in scale metrics between the JSS and MABEL items were addressed by rescaling the MABEL items to match the JSS range using a linear transformation (Müller‐Schneider 2022; Tarka 2015). The standardized dataset was then subjected to exploratory factor analysis (EFA) to identify underlying dimensions. Regression analyses explored associations between the 17‐item scale and participants' demographic and professional characteristics, identifying significant factors for deeper analysis.

### 2.2 Setting and Participant Recruitment

Participants for this study were recruited through the 2023 Australian Primary Health Care Nurses Association (APNA) Workforce Survey, a component of the annual nationwide Australian Primary Health Care (PHC) Nursing and Midwifery Workforce Survey, which has been conducted for over 15 years. The survey gathers data from a sample of the approximately 98 000 nurses and midwives across various PHC settings, including general practice, community health services, residential care facilities, correctional services, and Aboriginal and Torres Strait Islander health services.

The survey was disseminated through multiple channels, including the APNA homepage, targeted electronic messages sent to members and contacts, and posts on social media platforms such as Facebook, LinkedIn, and Instagram. Participants were also encouraged to share the survey with their networks of PHC nurses and midwives. In 2023, the survey garnered 2530 responses, including 1431 (56%) from GPNs.

### 2.3 Data Analyses

Descriptive statistics were used to summarize the demographic, professional, educational, and employment characteristics of GPNs. This analysis provided an overview of the participant group and established a context for subsequent exploratory analyses.

The mean, minimum, maximum, and standard deviation for each of the 17‐item scale questions were calculated to provide an overview of job satisfaction and workplace dynamics among GPN and midwives. This analysis offered insights into key aspects of their professional experiences and workplace environment.

EFA was conducted to identify the underlying dimensions of job satisfaction and work well‐being. Principal Axis Factoring with Varimax rotation and Kaiser Normalization was employed to extract meaningful patterns from the dataset. Cronbach's *α* was calculated to check the internal consistency of the questions in the 17‐item scale.

Binary logistic regression was performed to explore associations between job satisfaction and demographic factors. The enter method was used to assess predictors' contributions to outcomes such as age, years of nursing experience, employment status, and intentions to remain in the GPN role. Outcome variables were dichotomized to enhance analytical precision and minimize random error in non‐normally distributed data (Ferrazzo et al. 2012; MacCallum et al. 1999). Odds ratios (ORs) and 95% confidence intervals (95% CIs) quantified the strength and direction of associations.

All analyses were conducted using SPSS version 29, ensuring rigor in examining the relationships between job satisfaction, work well‐being, and demographic characteristics among GPNs.

## 3 Results

### 3.1 Descriptive Characteristics of GPN: Demographics, Education, and Employment

Table 1 shows that the GPN workforce is predominantly middle‐aged, with 56.9% of nurses aged 45–64 years. Females overwhelmingly represent the profession (97.3%), with minimal representation from males (2.7%) and non‐binary individuals (< 0.1%). Most nurses identify as non‐Indigenous Australians (97.9%), with a small percentage identifying as Aboriginal or Torres Strait Islander (2.1%). In terms of origin, 76.3% were born in Australia, while 2.7% were from New Zealand and 21.1% from other countries.

**TABLE 1:** Demographic, professional, educational, and employment characteristics of general practice nurses.

Category	Variable
Demographic characteristics	
Age group (*n* = 1427)	20–34 years—213 (14.9%); 35–44 years—272 (19.0%); 45–54 years—353 (24.7%); 55–64 years—461 (32.2%); 65+ years—128 (8.9%)
Sex (*n* = 1428)	Female—1389 (97.3%); Male—38 (2.7%); Non‐binary/gender diverse—1 (< 0.1%)
Aboriginal or Torres Strait Islander (*n* = 1417)	No—1387 (97.9%); Aboriginal—24 (1.7%); Torres Strait Islander—2 (0.1%); Other First Nations—4 (0.3%); Māori—1 (< 0.1%)
Country of birth (*n* = 1428)	Australia—1088 (76.3%); New Zealand—38 (2.7%); Other—302 (21.1%)
Professional background	
Nursing/midwifery qualification country (*n* = 1431)	Australia—1250 (87.35%); New Zealand—32 (2.24%); Other—149 (10.41%)
First nursing qualification (*n* = 1431)	Australia—1250 (87.3%); New Zealand—32 (2.2%); Other—149 (10.4%)
Years worked as nurse/midwife (*n* = 1417)	0–2 years—256 (17.9%); 3–9 years—500 (34.9%); 10–19 years—443 (31.0%); 20–29 years—160 (11.2%); 30+ years—58 (4.1%)
Years in PHC (*n* = 1417)	0–2 years—256 (18.07%); 3–9 years—500 (35.29%); 10–19 years—443 (31.26%); 20–29 years—160 (11.29%); 30+ years—58 (4.09%)
Education and specialization	
Highest education (*n* = 1422)	Certificate—140 (9.8%); Diploma—265 (18.6%); Undergraduate degree—454 (31.9%); Post‐grad certificate—272 (19.1%); Post‐grad diploma—174 (12.2%); Masters degree—116 (8.2%); Doctorate—1 (< 0.1%)
Specialty qualifications (*n* = 1230, multiple choice)	PHC/Health promotion—660 (37.37%); Admin/leadership—242 (13.7%); Chronic Disease Mgmt.—149 (8.44%); Diabetes—73 (4.13%); Education/teaching—81 (4.59%); Gerontology/aged care—35 (1.98%); Immunization—196 (11.1%); Infection control—105 (5.95%); Maternal/child health—37 (2.1%); Mental health—31 (1.76%); Palliative care—44 (2.49%); Research—11 (0.62%); WHS—57 (3.23%); Other—45 (2.55%)
Level of future studies (*n* = 688)	Certificate—218 (31.69%); Diploma—52 (7.56%); Undergraduate degree—58 (8.43%); Post‐grad certificate—167 (27.27%); Post‐grad diploma—62 (9.01%); Master's degree—116 (16.86%); Doctorate—15 (2.18%)
Employment and participation	
Participation in APNA TPP (*n* = 1421)	No—1317 (92.68%); Yes, partially completed—39 (2.74%); Yes, fully completed—65 (4.57%)
APNA mentor participation (*n* = 1418)	No—1359 (95.84%); Yes—59 (4.16%)
APNA student nurse placement (*n* = 1424)	No—1325 (93.05%); Unsure—57 (4.00%); Yes, general practice—10 (0.70%); Yes, aged care—32 (2.25%)
Primary health care placement (*n* = 1317)	No—804 (61.05%); Unsure—39 (2.96%); Yes—474 (35.99%)
Financial membership of APNA (*n* = 1427)	No—431 (30.20%); Yes—996 (69.80%)
Pay and workload	
Employment status (*n* = 1431)	Permanent—Full‐time—368 (25.7%); Permanent—Part‐time—749 (52.3%); Fixed‐term, Full‐time—5 (0.3%); Fixed‐term, Part‐time—25 (1.7%); Casual—269 (18.8%); Self‐employed—15 (1.0%)
Hourly pay (*n* = 1431)	0–30 AUD/h—71 (4.96%); 31–35 AUD/h—182 (12.72%); 36–40 AUD/h—406 (28.37%); 41–50 AUD/h—620 (43.33%); 51+ AUD/h—148 (10.34%)
Average weekly hours worked (*n* = 1431)	0–20 h—304 (21.24%); 21–35 h—738 (51.57%); 36–45 h—348 (24.32%); 46–120 h—40 (2.80%)
Secondary employment in PHC (*n* = 1414)	Yes—236 (16.69%); No—1178 (83.31%)
Intentions for future work	
Intention to commence further studies (*n* = 1419)	No—728 (51.27%); Yes, within 1 year—343 (24.15%); Yes, in 2–4 years—283 (19.93%); Yes, in 5 years or more—66 (4.65%)
Fields for future studies	Primary health care (including health promotion and public health)—25 (18.38%); Administration/leadership/management—11 (8.09%); Chronic disease management—19 (13.97%); Diabetes—11 (8.09%); Education/teaching—9 (6.62%); Gerontology/aged care—0 (0%); Immunization—12 (8.82%); Infection control—4 (2.94%); Maternal and child health—15 (11.03%); Mental health—7 (5.15%); Palliative/end‐of‐life care—8 (5.88%); Research—1 (0.74%); Work health and safety—1 (0.74%); Other field/s—13 (9.56%)
Intention to leave direct patient care (12 months) (*n* = 1287)	Likely—58 (4.51%); Very likely—73 (5.67%); Unsure—179 (13.91%); Unlikely—372 (28.90%); Very unlikely—603 (46.85%)
Intention to leave direct patient care (2–5 years) (*n* = 1298)	Likely—145 (11.17%); Very likely—126 (9.71%); Unsure—286 (22.03%); Unlikely—290 (22.34%); Very unlikely—451 (34.75%)
Intention to stay in PHC (12 months) (*n* = 1327)	Likely—266 (20.77%); Very likely—775 (60.50%); Unsure—127 (9.91%); Unlikely—39 (3.04%); Very unlikely—74 (5.78%)
Intention to stay in PHC (2–5 years) (*n* = 1296)	Likely—277 (21.37%); Very likely—536 (41.36%); Unsure—272 (20.99%); Unlikely—91 (7.02%); Very unlikely—120 (9.26%)
Intention to leave nursing (12 months) (*n* = 1287)	Likely—34 (2.64%); Very likely—55 (4.27%); Unsure—137 (10.64%); Unlikely—288 (22.38%); Very unlikely—773 (60.06%)
Intention to leave nursing (2–5 years) (*n* = 1300)	Likely—138 (10.62%); Very likely—129 (9.92%); Unsure—250 (19.28%); Unlikely—218 (16.77%); Very unlikely—565 (43.46%)

The majority of nurses obtained their qualifications in Australia (87.3%) and have substantial experience in the field, with 77% having worked for over 3 years and 31% for more than a decade. Similar trends are observed in GPNs, where 46.6% of nurses have over 10 years of experience (Table 1).

Table 1 also shows that educational backgrounds among GPNs were diverse, with undergraduate degrees (31.9%) and postgraduate certificates (19.1%) being the most common qualifications. Specialty qualifications in primary health care or health promotion (37.37%) were the most prevalent, followed by administration/leadership (13.7%) and immunization (11.1%). Interest in future studies was moderate, with 31.69% considering certificates and 16.86% master's degrees.

Employment patterns revealed that part‐time permanent positions dominate (52.3%), followed by full‐time permanent roles (25.7%). Hourly pay was primarily concentrated in the AUD 36–50 range (71.7%), and most nurses worked between 21 and 35 h weekly (51.57%). Secondary PHC employment was uncommon, with only 16.69% reporting additional roles in the sector.

Intentions to remain in direct patient care were high, with 75.75% planning to stay in the next 12 months and 57.09% over the next 2–5 years. Similarly, PHC retention intentions were strong, with 81.27% expecting to remain in the short term and 62.73% in the longer term (Table 1). Few nurses planned to leave nursing entirely, with only 6.91% considering it within 12 months and 20.54% over 2–5 years.

These findings emphasized a stable yet aging workforce with limited diversity. While short‐term retention in PHC and nursing appeared robust, long‐term sustainability requires attention. Workforce planning strategies should prioritize enhancing career pathways, education opportunities, and diversity to address emerging challenges (Table 1).

## 4 Job Satisfaction and Workplace Dynamics Among GPN and Midwives

The findings in Table 2 provide an overview of job satisfaction and workplace dynamics among nurses and midwives in primary health care (PHC), highlighting areas of strength and potential concerns. Overall job satisfaction is moderately high, with the mean rating for general satisfaction (“Taking everything into consideration, how do you feel about your work?”) at 5.23 (on a 6‐point scale). Satisfaction is highest with colleagues and fellow workers (mean = 5.51, SD = 1.044), physical working conditions (mean = 5.39, SD = 1.115), and hours of work (mean = 5.40, SD = 1.113). Nurses and midwives also report relatively high satisfaction with the variety in their work (mean = 5.34, SD = 1.134) and the amount of responsibility given (mean = 5.19, SD = 1.286). However, lower satisfaction is noted for recognition received for work (mean = 4.81, SD = 1.473) and remuneration (mean = 4.09, SD = 1.819), indicating areas for potential improvement.

**TABLE 2:** Satisfaction and agreement ratings among general practice nurses.

Question	Mean	Max	Min	SD	*N*
As a nurse or midwife in primary health care, how satisfied or dissatisfied you are with:					
Freedom to choose your own method of work	5.09	1	6	1.273	1332
Amount of variety in your work	5.34	1	6	1.134	1330
Physical working conditions	5.39	1	6	1.115	1330
Opportunities to use your abilities	5.02	1	6	1.384	1331
Your colleagues and fellow workers	5.51	1	6	1.044	1328
Recognition you get for your work	4.81	1	6	1.473	1335
Your hours of work	5.40	1	6	1.113	1330
Your remuneration	4.09	1	6	1.819	1334
Amount of responsibility you are given	5.19	1	6	1.286	1327
Taking everything into consideration, how do you feel about your work?	5.23	1	6	1.262	1330
In relation to your work as nurse or midwife in primary health care, how much you agree or disagree with:					
The balance between my personal and professional commitments is about right	3.96	1	5	1.007	1312
It is difficult to take time off work when I want to	2.78	1	5	1.265	1310
Communication among staff is encouraged and supported	3.99	1	5	1.078	1316
Multidisciplinary care planning and delivery is encouraged and supported_46	4.02	1	5	0.964	1316
I am able to consult with others about the management of patients/clients with complex health and social problems	4.26	1	5	0.872	1311
I have good support and supervision from qualified nurses/midwives or other health professionals	3.92	1	5	1.073	1282
I often undertake tasks that somebody less skilled or qualified could do	3.50	1	5	1.073	1300

Responses indicate mixed perceptions of workplace support and dynamics. Communication among staff is perceived positively (mean = 3.99, SD = 1.078), as is multidisciplinary care planning (mean = 4.02, SD = 0.964) and opportunities to consult about complex cases (mean = 4.26, SD = 0.872). However, support and supervision from qualified health professionals received a slightly lower rating (mean = 3.92, SD = 1.073), and the balance between personal and professional commitments is rated at 3.96 (SD = 1.007), suggesting moderate agreement.

Challenges emerge in areas such as the ability to take time off when desired (mean = 2.78, SD = 1.265), which highlights a potential issue with workplace flexibility. Additionally, the perception of often performing tasks suited for less skilled or qualified staff is rated at 3.50 (SD = 1.073), which may reflect inefficiencies in task delegation.

The data suggest a generally positive perception of work conditions and satisfaction with specific aspects such as teamwork, variety, and responsibility. However, areas such as remuneration, recognition, support, and work–life balance require attention to further enhance job satisfaction and workplace engagement among GPNs (Table 2). Addressing these areas could improve retention, performance, and overall morale in the workforce.

### 4.1 EFA of Job Satisfaction, Support, and Work–Life Balance Among GPN

#### 4.1.1 Dataset Suitability for Factor Analysis

The dataset's suitability for factor analysis was confirmed through factorial validity and reliability testing. Factorial validity, which evaluates the extent to which an instrument is free from measurement errors, was supported by a Kaiser–Meyer–Olkin (KMO) value of 0.941 and a *p* < 0.001 from Bartlett's test of sphericity, indicating that the sample was adequate for factor analysis. Additionally, reliability testing demonstrated strong internal consistency, with a Cronbach's *α* coefficient of 0.873 for the 17 positively worded scale items, further validating the dataset's appropriateness for this analytical approach.

The above KMO value and Cronbach's *α* further validate the coherence of the combined 17‐item scale, confirming its suitability for capturing the diverse dimensions of job satisfaction and work well‐being among GPNs.

#### 4.1.2 EFA Findings

EFA identified a three‐factor solution based on the eigenvalue > 1 criterion, explaining a cumulative 56.55% of the total variance (Table 3). Factor loadings ranged from 0.305 to 0.729, exceeding the threshold of 0.3, while the cumulative percentage of rotation sums of squared loadings ranged from 41.33% to 56.55%, underscoring the substantial contribution of these factors.

**TABLE 3:** Exploratory factor analysis result of work and wellbeing of general practice nurses.

Total variance explained									
Factor	Initial eigenvalues			Extraction sums of squared loadings			Rotation sums of squared loadings		
	Total	% of Variance	Cumulative %	Total	% of Variance	Cumulative %	Total	% of Variance	Cumulative %
1	7.028	41.344	41.344	6.554	38.550	38.550	4.024	23.671	23.671
2	1.360	8.001	49.345	0.891	5.239	43.789	2.607	15.337	39.009
3	1.226	7.209	56.554	0.583	3.427	47.216	1.395	8.207	47.216
4	0.945	5.560	62.115						
5	0.786	4.624	66.738						
6	0.687	4.041	70.780						
7	0.674	3.964	74.744						
8	0.551	3.241	77.985						
9	0.526	3.092	81.076						
10	0.493	2.897	83.974						
11	0.490	2.883	86.856						
12	0.455	2.677	89.533						
13	0.406	2.387	91.920						
14	0.377	2.217	94.137						
15	0.367	2.159	96.296						
16	0.342	2.010	98.306						
17	0.288	1.694	100.000						

Using Principal Axis Factoring as the extraction method, Factor 1 emerged as the most influential, with an initial eigenvalue of 7.028, accounting for 41.34% of the variance, which reduced to 23.67% after rotation. Factor 2, with an eigenvalue of 1.360, initially explained 8.00% of the variance, increasing to 15.34% post‐rotation, reflecting its role as a meaningful secondary dimension. Factor 3 had an eigenvalue of 1.226, contributing 7.21% initially and rising to 8.21% after rotation, representing another distinct but less prominent dimension. The remaining 14 factors, each with eigenvalues below 1, contributed minimally to the variance. Collectively, the three factors establish a robust structure, highlighting their significance in representing the underlying construct.

**FIGURE 1:**
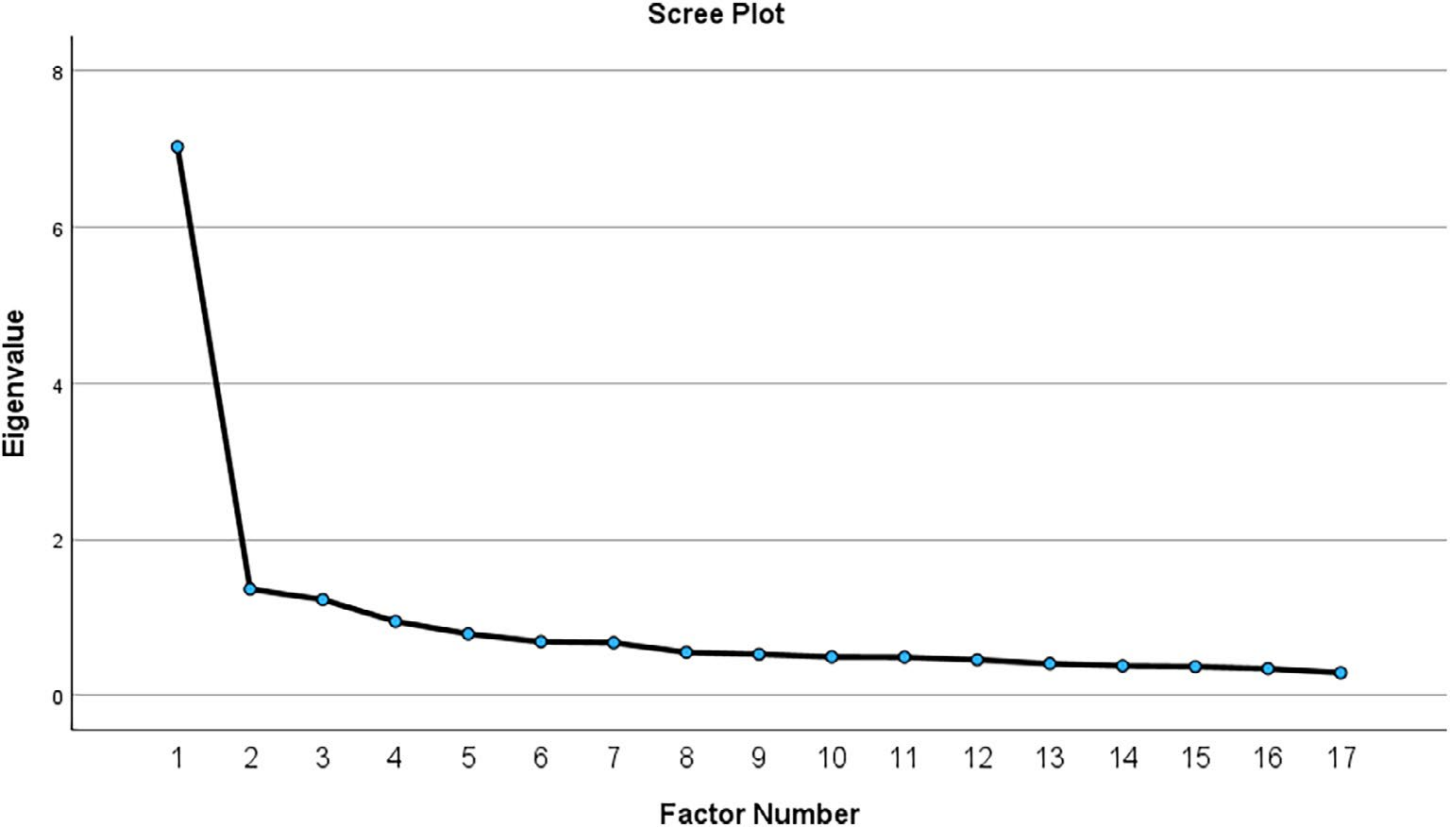
Scree plot to indicate the number of extracted factors.

The three‐factor solution was further validated by scree plots (Figure 1), where the curve leveled off after the third factor, visually confirming its appropriateness and alignment with the eigenvalue criterion. Subsequent factors contributed negligibly to the variance, as indicated by the flattened scree plot curve.

Table 4 identifies three underlying factors associated with job satisfaction and well‐being among GPNs, each with eigenvalues exceeding 1.

**TABLE 4:** Exploratory factor analysis of job satisfaction, support, and work–life balance among general practice nurses.

Factor 1: Job satisfaction and work environment	Item loading
As a nurse or midwife in primary health care, how satisfied or dissatisfied are you with opportunities to use your abilities	0.729
As a nurse or midwife in primary health care, how satisfied or dissatisfied are you with taking everything into consideration, how do you feel about your work?	0.704
As a nurse or midwife in primary health care, how satisfied or dissatisfied are you with amount of variety in your work	0.695
As a nurse or midwife in primary health care, how satisfied or dissatisfied are you with freedom to choose your own method of work	0.684
As a nurse or midwife in primary health care, how satisfied or dissatisfied are you with amount of responsibility you are given	0.659
As a nurse or midwife in primary health care, how satisfied or dissatisfied are you with physical working conditions	0.611
As a nurse or midwife in primary health care, how satisfied or dissatisfied are you with recognition you get for your work	0.563
As a nurse or midwife in primary health care, how satisfied or dissatisfied are you with your colleagues and fellow workers	0.498
As a nurse or midwife in primary health care, how satisfied or dissatisfied are you with your hours of work	0.426
As a nurse or midwife in primary health care, how satisfied or dissatisfied are you with your remuneration	0.423
Cronbach's *α*	0.899
Kaiser–Meyer–Olkin measure	0.938
Eigenvalue	1
% of variance	54.3

Factor 2: Support and communication	Item loading
As a nurse or midwife in primary health care, how satisfied or dissatisfied are you with recognition you get for your work	0.396
As a nurse or midwife in primary health care, how satisfied or dissatisfied are you with your colleagues and fellow workers	0.362
In relation to your work as nurse or midwife in primary health care, how much you agree or disagree with I am able to consult with others about the management of patients/clients with complex health and social problems_	0.746
In relation to your work as nurse or midwife in primary health care, how much you agree or disagree with Multidisciplinary care planning and delivery is encouraged and supported	0.663
In relation to your work as nurse or midwife in primary health care, how much you agree or disagree with Communication among staff is encouraged and supported_	0.625
In relation to your work as nurse or midwife in primary health care, how much you agree or disagree with I have good support and supervision from qualified nurses/midwives or other health professionals	0.613
Cronbach's *α*	0.833
Kaiser–Meyer–Olkin measure	0.861
Eigenvalue	1
% of variance	56.02

Factor 3: Work–life balance and work conditions	Item loading
As a nurse or midwife in primary health care, how satisfied or dissatisfied you are with Taking everything into consideration, how do you feel about your work?	0.356
As a nurse or midwife in primary health care, how satisfied or dissatisfied are you with recognition you get for your work	0.337
As a nurse or midwife in primary health care, how satisfied or dissatisfied are you with your hours of work	0.373
As a nurse or midwife in primary health care, how satisfied or dissatisfied are you with your remuneration	0.305
In relation to your work as nurse or midwife in primary health care, how much you agree or disagree with: the balance between my personal and professional commitments is about right	0.591
In relation to your work as nurse or midwife in primary health care, how much you agree or disagree with: It is difficult to take time off work when I want to	−0.5
Cronbach's *α*	0.625
Kaiser–Meyer–Olkin measure	0.805
Eigenvalue	1
% of variance	64.91

The first factor, Job Satisfaction and Work Environment, includes items related to abilities, job satisfaction, variety, autonomy, responsibility, conditions, and recognition, with loadings from 0.729 (abilities) to 0.423 (remuneration). These results underscore the importance of improving recognition, autonomy, and work conditions to enhance satisfaction.

The second factor, Support and Communication, involves multidisciplinary care, staff communication, supervisory support, and consultation, with loadings from 0.746 (consultation) to 0.362 (colleagues). This highlights the value of strong support systems and collaborative environments to boost well‐being.

The third factor, Work–Life Balance and Work Conditions, includes work–life balance, remuneration, and time off, with loadings from 0.591 (balance) to 0.305 (remuneration). The negative loading of −0.500 for “difficulty taking time off” indicates its adverse effect on balance. Policies promoting flexibility and fair compensation are crucial for retention and well‐being.

These three factors provide a comprehensive view of GPNs' job satisfaction and well‐being. Targeted interventions in these areas can enhance individual well‐being, workforce sustainability, and service delivery.

#### 4.1.3 Logistic Regression Analysis of Predictors for Key Demographic and Professional Outcomes in GPN

Table 5 highlights predictors of key demographic and professional outcomes in GPNs based on logistic regression analysis. Satisfaction with recognition increased the odds of being over 45 years old (OR = 1.202, *p* = 0.003) and satisfaction with work variety increased the odds of having over 10 years of experience (OR = 1.188, *p* = 0.034). Dissatisfaction with work–life balance reduced these odds by 21.2% (OR = 0.788, *p* = 0.004).

**TABLE 5:** Predictors for key demographic and professional outcomes in general practice nursing.

Demographic factor	Chi‐square	*p*	Nagelkerke *R* ^2^	*N*	Key findings based on odds ratio
Age (≤ 45 vs. > 45)	26.89	0.060	0.031	1171	Higher satisfaction with recognition for work increased the odds of being in the higher age group by 20.2% (OR = 1.202, *p* = 0.003).
Years of nursing experience (≤ 10 vs. > 10 years)	35.10	0.006	0.044	1151	Satisfaction with variety in work increased the odds of > 10 years of experience by 18.8% (OR = 1.188, *p* = 0.034), while dissatisfaction with work–life balance reduced the odds by 21.2% (OR = 0.788, *p* = 0.004).
Employment status (non‐permanent vs. permanent)	53.07	0.001	0.070	1164	Satisfaction with working hours increased the odds of permanent employment by 42.5% (OR = 1.425, *p* < 0.001), while dissatisfaction with recognition reduced the odds by 20.6% (OR = 0.820, *p* = 0.014).
Highest education (cert/diploma/undergrad vs. postgrad)	27.71	0.048	0.032	1167	Satisfaction with communication increased the odds of higher education by 19% (OR = 1.189, *p* = 0.025), while dissatisfaction with ability use reduced the odds by 19.1% (OR = 0.809, *p* = 0.001).
Intention to leave nursing (12 months)	53.41	0.001	0.124	1015	Overall work satisfaction reduced the odds of leaving nursing by 33.8% (OR = 0.662, *p* = 0.002).
Intention to leave nursing (2–5 years)	78.69	0.001	0.120	933	Dissatisfaction with work–life balance reduced the odds of leaving nursing by 27.6% (OR = 0.724, *p* < 0.001). Overall dissatisfaction reduced the odds by 21.8% (OR = 0.782, *p* = 0.007).
Hourly pay (up to $40 vs. > $40)	105.70	0.001	0.115	1173	Satisfaction with remuneration increased the odds of higher pay by 42.4% (OR = 1.424, *p* < 0.001), while good supervision reduced the odds by 18.7% (OR = 0.813, *p* = 0.008).
Intention to leave direct patient care (12 months)	52.24	0.001	0.102	973	Overall work satisfaction reduced the odds of leaving direct patient care by 27.7% (OR = 0.723, *p* = 0.007).
Intention to leave direct patient care (2–5 years)	68.87	0.001	0.109	892	Key predictors included dissatisfaction with work–life balance (OR = 0.769, *p* = 0.008) and overall satisfaction (OR = 0.781, *p* = 0.007).
Intention to leave PHC (12 months)	100.07	0.001	0.200	1007	Higher job satisfaction reduced the odds of leaving by 51.1% (OR = 0.489, *p* < 0.001).
Intention to leave PHC (2–5 years)	131.52	0.001	0.211	908	Job satisfaction reduced the odds of leaving by 48.6% (OR = 0.514, *p* < 0.001), while satisfaction with colleagues increased the odds by 26.2% (OR = 1.262, *p* = 0.027).
Country of birth (Australia vs. other)	25.96	0.075	0.033	1173	Dissatisfaction with ability use reduced the odds of being born outside Australia by 14.1% (OR = 0.859, *p* = 0.040).
Years in PHC (< 10 vs. ≥ 10 years)	30.95	0.020	0.035	1165	Dissatisfaction with work–life balance reduced the odds of ≥ 10 years in PHC by 17.9% (OR = 0.821, *p* = 0.005).
Average weekly hours (full‐time vs. part‐time)	63.58	0.001	0.074	1173	Satisfaction with variety in work increased the odds of part‐time work by 19.6% (OR = 1.196, *p* = 0.032), while dissatisfaction with work–life balance reduced the odds by 26.6% (OR = 0.734, *p* < 0.001).
Primary health care placement (yes vs. no)	18.81	0.339	0.066	1127	Satisfaction with working hours increased the odds of PHC placement by 23.5% (OR = 1.235, *p* = 0.194), while satisfaction with work–life balance increased the odds by 23.4% (OR = 1.234, *p* = 0.251).
APNA membership (yes vs. no)	21.75	0.195	0.026	1171	Satisfaction with ability use increased the odds of membership by 22.8% (OR = 1.228, *p* = 0.005).
Intention to commence studies (yes vs. no)	34.01	0.008	0.038	1169	Satisfaction with ability use increased the odds of further studies by 27.6% (OR = 1.276, *p* < 0.001).
Level of future studies (entry vs. advanced)	26.15	0.072	0.059	581	Satisfaction with communication increased the odds of advanced qualifications by 28.3% (OR = 1.283, *p* = 0.020), while dissatisfaction with recognition reduced the odds by 17.3% (OR = 0.827, *p* = 0.028).

Satisfaction with working hours increased the odds of permanent employment (OR = 1.425, *p* < 0.001), while dissatisfaction with recognition lowered them (OR = 0.820, *p* = 0.014). Satisfaction with communication was associated with higher education levels (OR = 1.189, *p* = 0.025), but dissatisfaction with ability use reduced the odds (OR = 0.809, *p* = 0.001). Overall job satisfaction reduced the odds of leaving nursing within 12 months (OR = 0.662, *p* = 0.002) and 2–5 years (OR = 0.782, *p* = 0.007). Dissatisfaction with work–life balance further decreased the odds of leaving over 2–5 years (OR = 0.724, *p* < 0.001). Satisfaction reduced the likelihood of leaving direct patient care in the short (OR = 0.723, *p* = 0.007) and long term (OR = 0.781, *p* = 0.007), and strongly influenced PHC retention within 12 months (OR = 0.489, *p* < 0.001) and 2–5 years (OR = 0.514, *p* < 0.001).

Satisfaction with remuneration increased the odds of earning over $40/h (OR = 1.424, *p* < 0.001), while dissatisfaction with work–life balance reduced the likelihood of part‐time work (OR = 0.734, *p* < 0.001). Satisfaction with ability use increased the odds of pursuing further studies (OR = 1.276, *p* < 0.001) and advanced qualifications (OR = 1.283, *p* = 0.020), while dissatisfaction with recognition lowered these odds (OR = 0.827, *p* = 0.028). Satisfaction with ability use also increased APNA membership (OR = 1.228, *p* = 0.005).

These findings highlight the impact of job satisfaction on critical outcomes, including retention, career progression, and workplace preferences. Enhancing satisfaction, particularly in recognition, work–life balance, and remuneration, could improve workforce sustainability and engagement in GPNs.

## 5 Discussion

This study highlights key insights into the GPN workforce in Australia, emphasizing the demographic composition, professional experiences, and challenges faced by this predominantly female workforce.

### 5.1 Demographic Insights and Workforce Characteristics

The data provide valuable insights into the GPN workforce, shedding light on key characteristics such as age distribution, professional experience, and diversity. Over half of the workforce is aged 45–64, with the largest proportion (32.2%) in the 55–64 age group. Additionally, 35.9% of respondents reported over 30 years of nursing experience. While this highlights a wealth of expertise, it also raises significant concerns about an aging workforce, with many nurses nearing retirement age (APNA 2025b). The underrepresentation of younger nurses, particularly those under 35, highlights the challenges in attracting new entrants to general practice nursing, despite the increasing demand for primary care services. This generational gap calls for targeted recruitment and retention initiatives to ensure a sustainable workforce.

The Australian Government Department of Health and Aged Care maintains a Health Workforce Data Tool (Commonwealth of Australia 2023), which provides insights into the nursing workforce. According to this tool, 31.30% of nurses were aged 20–34, a significantly higher proportion than in our dataset (14.88%). Conversely, the 55–64 age group showed the opposite trend, with 21.06% in the Health Workforce Data Tool compared to 32.22% in our dataset. This skewed age distribution resulted in a higher mean age for GPNs in our dataset compared to the Health Workforce Data Tool. Caution is needed when interpreting findings and making policy decisions based on age in our dataset.

Cultural diversity within the GPN workforce remains limited, with only 2.1% of respondents identifying as Aboriginal or Torres Strait Islander and overall minority representation remaining low. This lack of diversity is particularly concerning given the multicultural nature of many communities served by GPNs, potentially limiting the provision of culturally sensitive care (Shepherd et al. 2019). Enhancing diversity within the workforce is essential to addressing health inequities and improving outcomes for culturally and linguistically diverse populations. This requires proactive strategies, including targeted recruitment, culturally sensitive training, and support programs for underrepresented groups.

A significant proportion of GPNs (51.3%) reported no intention to pursue further education, despite their extensive experience. This reluctance highlights systemic barriers to professional development, such as financial constraints, time limitations, and limited institutional support. (Parker et al. 2011) Employment trends reveal a strong preference for part‐time roles (52.3%), reflecting the life stage and personal commitments of many GPNs (Komagata et al. 2020). Moderate levels of job satisfaction, with a mean score of 48.7 out of 67, are consistent with previous findings (Halcomb and Ashley 2017; Kim et al. 2024) but emphasize room for improvement, particularly in addressing workplace challenges and enhancing professional growth opportunities.

### 5.2 Job Satisfaction and Work Environment

The factor analysis identified job satisfaction and the work environment as critical dimensions influencing GPNs' well‐being. Opportunities to use abilities, variety in work, and autonomy were the strongest contributors to satisfaction, emphasizing their central role in fostering a positive workplace environment. Conversely, remuneration and working hours emerged as persistent challenges, with lower factor loadings reflecting dissatisfaction in these areas. These findings align with existing literature, which links dissatisfaction with pay and work conditions to increased turnover intentions among healthcare professionals (Senek et al. 2020; Bimpong et al. 2020).

Remuneration remains a particularly contentious issue for GPNs, compounded by fluctuating federal policies and inconsistent Medicare funding models (Heywood and Laurence 2018). The Scope of Practice review has proposed recommendations such as multidisciplinary combined payment provider numbers to address funding gaps (Cormack 2024). However, these remain at the policy recommendation stage and lack widespread implementation (Cormack 2024). Expanding the scope of practice and fostering collaborative opportunities within multidisciplinary teams are critical steps toward resolving these systemic issues (Cormack 2024). Ensuring adequate pay structures and career advancement pathways will be essential for retaining skilled nurses and attracting new talent.

Similar workforce challenges have been reported internationally, reinforcing the broader relevance of our findings. In the United Kingdom, for example, the general practice nursing workforce is also aging, with 44% of practice nurses aged over 50 years, raising similar concerns about succession planning and workforce sustainability (Lewis 2024; Qicn 2025). Likewise, in Canada, primary care nurses have expressed concerns about limited opportunities for career advancement, inconsistent funding models, and insufficient role clarity, all of which mirror the barriers faced by Australian GPNs (Brault et al. 2014; Donald et al. 2010). In the United States, job satisfaction among nurses in primary care is closely tied to scope of practice, remuneration, and supportive work environments, factors that are consistent with our regression findings (Hoff et al. 2019). These parallels suggest that Australia's challenges are not unique, and that solutions such as population‐based funding models, role expansion, and structured professional development, which were successfully piloted in parts of the United Kingdom and United States of America, may offer valuable insights for strengthening the Australian GPN workforce, although a recent review commissioned as part of the Scope of Practice Review found mixed results (Kidd et al. 2024).

### 5.3 Work–Life Balance and Professional Development

Work–life balance was identified as a key factor influencing GPNs' well‐being. The negative loading for the item “It is difficult to take time off work when I want to” underscores the challenges many GPNs face in balancing professional responsibilities with personal commitments. These challenges are particularly acute in a predominantly female workforce, where family obligations often take precedence (Workplace Gender Equality Agency 2016). This likely contributes to the high prevalence of part‐time employment and highlights the need for policies that support flexible working arrangements and family‐friendly environments.

Barriers to professional development continue to limit the ability of GPNs to progress in their careers. This challenge is compounded by the private business structure of general practice in Australia, where educational support is often deprioritized, and by inconsistent government funding mechanisms that fail to provide adequate resources for continuing education (Pearce et al. 2011). While university programs tend to offer broad primary care content, they often overlook the specific competencies required in general practice nursing. These competencies include the ability to deliver person‐centred care across the lifespan, ranging from acute care to chronic disease management, and from infants to older adults, within diverse cultural contexts. GPNs are also expected to support team‐based, collaborative care models, engage in quality improvement initiatives, contribute to practice accreditation processes, and navigate digital health systems (Halcomb et al. 2017). Furthermore, financial literacy is increasingly valued, with GPNs playing a role in maximizing practice incentives through knowledge of funding models and Medicare item usage. Despite their importance, these multifaceted skills are often underemphasized in mainstream curricula and require targeted, practice‐specific education and support (Pascoe et al. 2007).

To overcome these limitations, policy reforms are needed to secure stable, dedicated funding streams that subsidize continuing professional development (CPD) for GPNs (Main and Anderson 2023). In addition, the development of accredited graduate certificate programs specifically designed for general practice nursing would ensure that education pathways align with the role's demands (Main and Anderson 2023; Department of Health and Aged Care 2025). Employers also require support to implement protected learning time, allowing nurses to pursue CPD without negatively impacting patient care or personal wellbeing (RACGP 2025). Together, these measures would promote professional growth, improve job satisfaction, and strengthen workforce retention.

At the same time, improving cultural diversity within the GPN workforce demands deliberate and sustained action. Recruitment strategies should include culturally inclusive initiatives aimed at increasing representation from Aboriginal and Torres Strait Islander populations and other CALD backgrounds (Commonwealth of Australia 2024; Department of Health 2022). These strategies could involve offering scholarships, establishing bridging programs, and building partnerships with Indigenous nursing organizations. Retention can also be improved through structured mentorship programs and mandatory training in culturally safe and responsive care. Such efforts are vital not only to promote workforce equity but also to ensure culturally safe care delivery in Australia's increasingly diverse primary healthcare landscape (Biles et al. 2024).

### 5.4 Demographic Predictors of Job Satisfaction and Retention

Logistic regression analysis revealed strong associations between demographic factors and job satisfaction. Employment status, hourly pay, and intentions to leave nursing were the most significant predictors. Non‐permanent employment was associated with lower satisfaction, highlighting the importance of job security. Dissatisfaction with pay and limited career advancement opportunities were key drivers of turnover intentions, reinforcing the need for competitive remuneration structures and expanded career pathways.

To ensure workforce sustainability, two priorities emerge: establishing a reliable funding source for GPNs and enabling them to work to their full scope of practice. Lessons from international models, such as the UK's Additional Roles and Remuneration Scheme, demonstrate the potential benefits of population‐based funding models in enhancing workforce stability (Eaton et al. 2020; Gibson et al. 2023). In Australia, however, the fee‐for‐service model remains dominant, and recent reviews of general practice funding have not supported significant shifts in this approach (Kidd et al. 2024). Exploring alternative funding mechanisms will be critical for addressing the long‐term sustainability of the GPN workforce.

### 5.5 Study Strengths and Limitations

This study has several limitations. Capturing sufficient numbers of nurses working in general practice was challenging, potentially limiting the generalizability of findings (Halcomb and Ashley 2017; Halcomb et al. 2021). The predominance of older, experienced respondents may exclude the perspectives of younger nurses, although this reflects the current workforce demographic. Reliance on self‐reported data introduces potential biases, such as social desirability and recall inaccuracies.

Despite these limitations, the study offers notable strengths. The large sample size (*n* = 1431) enabled robust analyses of demographic and professional trends. The inclusion of comprehensive variables, such as registration status, age, gender, education level, and work experience, facilitated an in‐depth workforce analysis. The significant experience of respondents, with over one‐third having more than 30 years in nursing, provides valuable insights into a skilled and mature workforce. Additionally, the study's focus on job satisfaction and professional development offers actionable insights for workforce planning and retention strategies. Future research should adopt longitudinal and qualitative approaches to provide deeper insights into workforce dynamics and address the identified gaps in GPN experiences.

## 6 Conclusion

This study identified key factors influencing job satisfaction, retention, and workforce sustainability among GPNs in Australia. It highlights challenges such as an aging workforce, limited diversity, and barriers to professional development, alongside opportunities to enhance job satisfaction through improved remuneration, expanded scope of practice, and supportive work environments. Addressing these factors through targeted policies and workforce planning is essential to sustaining a skilled and equitable GPN workforce, ensuring their vital role in meeting Australia's evolving primary care needs.

## 7 Clinical Practice and Policy Implications

This study highlights strategies to address challenges in the GPN workforce. Clinically, improving job satisfaction through better work environments, recognition, and flexible scheduling can support retention. Promoting tailored professional development ensures GPNs remain skilled for evolving primary care needs.

Policy priorities include stable funding for remuneration and education, expanding GPNs' scope of practice, and incentivizing diverse recruitment to improve culturally sensitive care. Exploring population‐based funding models may enhance workforce sustainability. These measures are crucial for maintaining a robust GPN workforce to meet Australia's primary care demands.

## Author Contributions

**Wenpeng You:** conceptualization, investigation, funding acquisition, writing – original draft, methodology, validation, visualization, writing – review and editing, software, formal analysis, project administration, data curation, resources. **Claire Verrall:** conceptualization, investigation, funding acquisition, methodology, validation, visualization, writing – review and editing, data curation, resources. **Eileen Willis:** conceptualization, investigation, funding acquisition, writing – review and editing, visualization, validation, project administration, resources. **Danny Hills:** investigation, conceptualization, funding acquisition, methodology, validation, visualization, writing – review and editing, software, project administration, resources, data curation.

## Ethics Statement

Ethical approval for the conduct of the APNA Workforce Survey was obtained from Monash University (39381). For the current sub‐study, ethical approval was additionally obtained from the University of Adelaide (H‐2023‐129).

## Conflicts of Interest

The authors declare no conflicts of interest.

## Data Availability

The data that support the findings of this study are available from the corresponding author upon reasonable request.

## References

[nhs70130-bib-0001] Ahpra . 2024. “Fact Sheet: Continuing Professional Development.” https://www.nursingmidwiferyboard.gov.au/codes‐guidelines‐statements/faq/cpd‐faq‐for‐nurses‐and‐midwives.aspx.

[nhs70130-bib-0002] Alkan, E. , N. Cushen‐Brewster , and P. Anyanwu . 2024. “Organisational Factors Associated With Healthcare Workforce Development, Recruitment, and Retention in the United Kingdom: A Systematic Review.” BMC Nursing 23, no. 1: 604. 39217386 10.1186/s12912-024-02216-0 PMC11366130

[nhs70130-bib-0003] APNA . 2024. “Annual APNA Workforce Survey 2023: Insights for Advocacy and Change.” https://www.apna.asn.au/hub/primary‐times‐articles/primary‐times‐winter‐2024/insights‐for‐advocacy‐and‐change.

[nhs70130-bib-0004] APNA . 2025a. “APNA Career and Education Frameworks for Nurses in Primary Health Care.” https://www.apna.asn.au/nursing‐tools/framework.

[nhs70130-bib-0005] APNA . 2025b. “Transition to Practice Programs.” https://www.apna.asn.au/education/TransitiontoPracticeProgram.

[nhs70130-bib-0006] Biles, B. , B. Christian , C. Marshall , et al. 2024. “‘DANMM That's Good!’: Evaluating the Feasibility and Acceptability of the Deadly Aboriginal and Torres Strait Islander Nursing and Midwifery Mentoring (DANMM) Programme Across Rural, Regional and Metropolitan NSW—A Collaborative Study Protocol.” BMJ Open 14, no. 2: e079416. 10.1136/bmjopen-2023-079416 PMC10862277 38341205

[nhs70130-bib-0007] Bimpong, K. A. A. , A. Khan , R. Slight , C. L. Tolley , and S. P. Slight . 2020. “Relationship Between Labour Force Satisfaction, Wages and Retention Within the UK National Health Service: A Systematic Review of the Literature.” BMJ Open 10, no. 7: e034919. 10.1136/bmjopen-2019-034919 PMC7375434 32699127

[nhs70130-bib-0008] Brault, I. , K. Kilpatrick , D. D'Amour , et al. 2014. “Role Clarification Processes for Better Integration of Nurse Practitioners Into Primary Healthcare Teams: A Multiple‐Case Study.” Nursing Research and Practice 2014, no. 1: 170514. 25692039 10.1155/2014/170514 PMC4322308

[nhs70130-bib-0009] Chenowethm, L. , Y. H. Jeon , M. Goff , and C. Burke . 2006. “Cultural Competency and Nursing Care: An Australian Perspective.” International Nursing Review 53, no. 1: 34–40. 16430758 10.1111/j.1466-7657.2006.00441.x

[nhs70130-bib-0010] Commonwealth of Australia . 2023. “Health Workforce Data.” https://hwd.health.gov.au/datatool/.

[nhs70130-bib-0011] Commonwealth of Australia . 2024. “Aboriginal and Torres Strait Islander Recruitment Information.”

[nhs70130-bib-0012] Cormack, M. 2024. “Unleashing the Potential of Our Health Workforce—Scope of Practice Review.” https://www.health.gov.au/resources/publications/unleashing‐the‐potential‐of‐our‐health‐workforce‐scope‐of‐practice‐review‐final‐report.

[nhs70130-bib-0013] Department of Health . 2022. “National Aboriginal and Torres Strait Islander Health Workforce Strategic Framework and Implementation Plan (2021–2031).” https://www.health.gov.au/sites/default/files/documents/2022/03/national‐aboriginal‐and‐torres‐strait‐islander‐health‐workforce‐strategic‐framework‐and‐implementation‐plan‐2021‐2031.pdf?utm_source=chatgpt.com.

[nhs70130-bib-0014] Department of Health and Aged Care . 2025. “Workforce Incentive Program (WIP)—Practice Stream.” https://www.health.gov.au/our‐work/workforce‐incentive‐program/practice‐stream?utm_source=chatgpt.com.

[nhs70130-bib-0015] Donald, F. , R. Martin‐Misener , D. Bryant‐Lukosius , et al. 2010. “The Primary Healthcare Nurse Practitioner Role in Canada.” Nursing Leadership (Toronto, Ont.) 23: 88–113. 21478689 10.12927/cjnl.2013.22271

[nhs70130-bib-0016] Eaton, G. , G. Wong , V. Williams , N. Roberts , and K. R. Mahtani . 2020. “Contribution of Paramedics in Primary and Urgent Care: A Systematic Review.” British Journal of General Practice 70, no. 695: e421–e426. 10.3399/bjgp20X709877 PMC7239041 32424047

[nhs70130-bib-0017] Ferrazzo, M. , M. Filippi , G. Meneghetti , and A. Palese . 2012. “Determinants of the Choice of Part Time Employment and Nurses' Satisfaction: A Multicentre Descriptive Study.” Assistenza Infermieristica e Ricerca: AIR 31, no. 4: 207–213. 23334641 10.1702/1211.13409

[nhs70130-bib-0018] Galanis, P. , I. Moisoglou , M. Malliarou , et al. 2023. “Quiet Quitting Among Nurses Increases Their Turnover Intention: Evidence From Greece in the Post‐COVID‐19 Era.” In Healthcare. MDPI. 10.3390/healthcare12010079 PMC10779139 38200985

[nhs70130-bib-0019] Gibson, J. , A. McBride , K. Checkland , et al. 2023. “General Practice Managers' Motivations for Skill Mix Change in Primary Care: Results From a Cross‐Sectional Survey in England.” Journal of Health Services Research & Policy 28, no. 1: 5–13. 35977066 10.1177/13558196221117647 PMC9850398

[nhs70130-bib-0020] Halcomb, E. , and C. Ashley . 2017. “Australian Primary Health Care Nurses Most and Least Satisfying Aspects of Work.” Journal of Clinical Nursing 26, no. 3–4: 535–545. 27461981 10.1111/jocn.13479

[nhs70130-bib-0021] Halcomb, E. , and C. Ashley . 2019. “Are Australian General Practice Nurses Underutilised?: An Examination of Current Roles and Task Satisfaction.” Collegian 26, no. 5: 522–527.

[nhs70130-bib-0022] Halcomb, E. , and S. Bird . 2020. “Job Satisfaction and Career Intention of Australian General Practice Nurses: A Cross‐Sectional Survey.” Journal of Nursing Scholarship 52, no. 3: 270–280. 32187812 10.1111/jnu.12548

[nhs70130-bib-0023] Halcomb, E. , S. Bird , S. Mcinnes , C. Ashley , and K. Huckel . 2021. “Exploring Job Satisfaction and Turnover Intentions Among General Practice Nurses in an Australian Primary Health Network.” Journal of Nursing Management 29, no. 5: 943–952. 33306862 10.1111/jonm.13230

[nhs70130-bib-0024] Halcomb, E. , M. Stephens , J. Bryce , E. Foley , and C. Ashley . 2017. “The Development of Professional Practice Standards for Australian General Practice Nurses.” Journal of Advanced Nursing 73, no. 8: 1958–1969. 28181277 10.1111/jan.13274

[nhs70130-bib-0025] Heywood, T. , and C. Laurence . 2018. “The General Practice Nurse Workforce:'Estimating Future Supply'.” Australian Journal of General Practice 47, no. 11: 788–795. 31207678 10.31128/AJGP-01-18-4461

[nhs70130-bib-0026] Hills, D. , C. Joyce , and J. Humphreys . 2012. “Validation of a Job Satisfaction Scale in the Australian Clinical Medical Workforce.” Evaluation & the Health Professions 35, no. 1: 47–76. 21411473 10.1177/0163278710397339

[nhs70130-bib-0027] Hoff, T. , S. Carabetta , and G. E. Collinson . 2019. “Satisfaction, Burnout, and Turnover Among Nurse Practitioners and Physician Assistants: A Review of the Empirical Literature.” Medical Care Research and Review 76, no. 1: 3–31. 28901205 10.1177/1077558717730157

[nhs70130-bib-0028] Joyce, C. M. , A. Scott , S. H. Jeon , et al. 2010. “The ‘Medicine in Australia: Balancing Employment and Life (MABEL)’ Longitudinal Survey‐Protocol and Baseline Data for a Prospective Cohort Study of Australian Doctors' Workforce Participation.” BMC Health Services Research 10: 1–10. 20181288 10.1186/1472-6963-10-50 PMC2837653

[nhs70130-bib-0029] Kamau, S. , M. Koskenranta , H. Kuivila , et al. 2022. “Integration Strategies and Models to Support Transition and Adaptation of Culturally and Linguistically Diverse Nursing Staff Into Healthcare Environments: An Umbrella Review.” International Journal of Nursing Studies 136: 104377. 36327682 10.1016/j.ijnurstu.2022.104377

[nhs70130-bib-0030] Khatri, R. B. , and Y. Assefa . 2022. “Access to Health Services Among Culturally and Linguistically Diverse Populations in the Australian Universal Health Care System: Issues and Challenges.” BMC Public Health 22, no. 1: 880. 35505307 10.1186/s12889-022-13256-z PMC9063872

[nhs70130-bib-0031] Kidd, M. , J. Rhee , A. Sharma , et al. 2024. “Review of General Practice Incentives: International Evidence and Literature Review, Commissioned by: Australian Department of Health and Aged Care.” https://www.health.gov.au/sites/default/files/2024‐10/review‐of‐general‐practice‐incentives‐international‐evidence‐and‐literature‐review.docx.

[nhs70130-bib-0032] Kim, E. , H. Kim , and T. Lee . 2024. “How Are New Nurses Satisfied With Their Jobs? From the Work Value Perspective of Generations Y and Z Nurses.” BMC Nursing 23, no. 1: 252. 38643129 10.1186/s12912-024-01928-7 PMC11032593

[nhs70130-bib-0033] Komagata, M. , Y. Takemura , N. Ichikawa , K. Takehara , and K. Kunie . 2020. “Quality of Work Among Part‐Time Nurses and Its Relationship to Job Satisfaction and Work Values: A Cross‐Sectional Study.” Nursing & Health Sciences 22, no. 4: 1010–1021. 32677152 10.1111/nhs.12760

[nhs70130-bib-0034] Lewis, R. 2024. “Developing a ‘National Module’ for Nurses Considering a Career in General Practice: Addressing the Workforce Crisis in Primary Care.” Practice Nursing 35, no. 4: 136–139.

[nhs70130-bib-0035] MABEL . 2024. “Medicine in Australia: Balancing Employment and Life Australia's national longitudinal survey of doctors (MABEL).” https://melbourneinstitute.unimelb.edu.au/mabel.

[nhs70130-bib-0036] MacAskill, W. , R. M. Rolleston , K. Brumpton , and J. Pinidiyapathirage . 2022. “Assessing Health Literacy of Aboriginal and Torres Strait Islander Peoples Presenting to General Practice.” Australian Journal of General Practice 51, no. 8: 621–625. 35908756 10.31128/AJGP-07-21-6100

[nhs70130-bib-0037] MacCallum, R. C. , K. F. Widaman , S. Zhang , and S. Hong . 1999. “Sample Size in Factor Analysis.” Psychological Methods 4, no. 1: 84–99.

[nhs70130-bib-0038] Maheen, H. , K. Chalmers , S. Khaw , and C. McMichael . 2021. “Sexual and Reproductive Health Service Utilisation of Adolescents and Young People From Migrant and Refugee Backgrounds in High‐Income Settings: A Qualitative Evidence Synthesis (QES).” Sexual Health 18, no. 4: 283–293. 34412768 10.1071/SH20112

[nhs70130-bib-0039] Main, P. A. E. , and S. Anderson . 2023. “Evidence for Continuing Professional Development Standards for Regulated Health Practitioners in Australia: A Systematic Review.” Human Resources for Health 21, no. 1: 23. 36941655 10.1186/s12960-023-00803-x PMC10026429

[nhs70130-bib-0040] McKenna, L. , E. Halcomb , R. Lane , N. Zwar , and G. Russell . 2015. “An Investigation of Barriers and Enablers to Advanced Nursing Roles in Australian General Practice.” Collegian 22, no. 2: 183–189. 26281406 10.1016/j.colegn.2015.02.003

[nhs70130-bib-0042] Mlambo, M. , C. Silén , and C. McGrath . 2021. “Lifelong Learning and Nurses' Continuing Professional Development, a Metasynthesis of the Literature.” BMC Nursing 20: 1–13. 33853599 10.1186/s12912-021-00579-2 PMC8045269

[nhs70130-bib-0043] Müller‐Schneider, T. 2022. “Exploratory Likert Scaling as an Alternative to Exploratory Factor Analysis: Methodological Foundation and a Comparative Example Using an Innovative Scaling Procedure.” Methods, Data, Analyses: A Journal for Quantitative Methods and Survey Methodology (MDA) 16, no. 1: 51–76.

[nhs70130-bib-0044] Nicole Zonin, M. 2022. “Older Nurses' Perceptions of Workforce Retention Facilitators and Barriers During the COVID‐19 Pandemic.” Online Journal of Issues in Nursing 27, no. 3: 1–12.

[nhs70130-bib-0045] Papp, M. , L. Kőrösi , J. Sándor , C. Nagy , A. Juhász , and R. Ádány . 2019. “Workforce Crisis in Primary Healthcare Worldwide: Hungarian Example in a Longitudinal Follow‐Up Study.” BMJ Open 9, no. 7: e024957. 10.1136/bmjopen-2018-024957 PMC6661691 31340955

[nhs70130-bib-0046] Parker, R. , H. Keleher , and L. Forrest . 2011. “The Work, Education and Career Pathways of Nurses in Australian General Practice.” Australian Journal of Primary Health 17, no. 3: 227–232. 21896258 10.1071/PY10074

[nhs70130-bib-0047] Pascoe, T. , R. Hutchinson , E. Foley , I. Watts , L. Whitecross , and T. Snowdon . 2007. “The Educational Needs of Nurses Working in Australian General Practices.” Australian Journal of Advanced Nursing 24, no. 3: 33–37. 17518163

[nhs70130-bib-0048] Pearce, C. , C. Phillips , S. Hall , et al. 2011. “Following the Funding Trail: Financing, Nurses and Teamwork in Australian General Practice.” BMC Health Services Research 11: 1–9. 21329506 10.1186/1472-6963-11-38 PMC3050696

[nhs70130-bib-0049] Qicn . 2025. “General Practice Nursing in the 21st Century: A Time of Opportunity.” https://qicn.org.uk/resources/general‐practice‐nursing‐21st‐century/?utm_source=chatgpt.com.

[nhs70130-bib-0050] RACGP . 2025. “CPD Activities Linked to Government Initiatives or Funding—General Practice Mental Health Standards Collaboration.” https://www.racgp.org.au/education/professional‐development/continuing‐professional‐development‐cpd‐program/cpd‐activities‐linked‐to‐government‐initiatives‐or?utm_source=chatgpt.com.

[nhs70130-bib-0051] Reid, M. , and S. Knight . 2024. “Working Better for Medicare Review.”

[nhs70130-bib-0052] Senek, M. , S. Robertson , T. Ryan , et al. 2020. “Determinants of Nurse Job Dissatisfaction—Findings From a Cross‐Sectional Survey Analysis in the UK.” BMC Nursing 19: 1–10. 32963498 10.1186/s12912-020-00481-3 PMC7499408

[nhs70130-bib-0053] Shepherd, S. M. , C. Willis‐Esqueda , D. Newton , D. Sivasubramaniam , and Y. Paradies . 2019. “The Challenge of Cultural Competence in the Workplace: Perspectives of Healthcare Providers.” BMC Health Services Research 19: 1–11. 30808355 10.1186/s12913-019-3959-7 PMC6390600

[nhs70130-bib-0054] Skillman, D. , and R. Toms . 2022. “Factors Influencing Nurse Intent to Leave Acute Care Hospitals: A Systematic Literature Review.” JONA: The Journal of Nursing Administration 52, no. 12: 640–645. 10.1097/NNA.0000000000001225 36409256

[nhs70130-bib-0055] Tarka, P. 2015. “Likert Scale and Change in Range of Response Categories vs. the Factors Extraction in EFA Model.” Acta Universitatis Lodziensis. Folia Oeconomica 1, no. 311: 27–36.

[nhs70130-bib-0056] Verrall, C. , E. Willis , and J. Henderson . 2023. “Practice Nursing: A Systematic Literature Review of Facilitators and Barriers in Three Countries.” Collegian 30, no. 2: 254–263.

[nhs70130-bib-0057] Walter, J. K. , and L. M. Terry . 2021. “Factors Influencing Nurses' Engagement With CPD Activities: A Systematic Review.” British Journal of Nursing 30, no. 1: 60–68. 33433281 10.12968/bjon.2021.30.1.60

[nhs70130-bib-0058] Workplace Gender Equality Agency . 2016. “Unpaid Care Work and the Labour Market: Insight Paper.” https://www.wgea.gov.au/sites/default/files/documents/australian‐unpaid‐care‐work‐and‐the‐labour‐market.pdf?utm_source=chatgpt.com.

